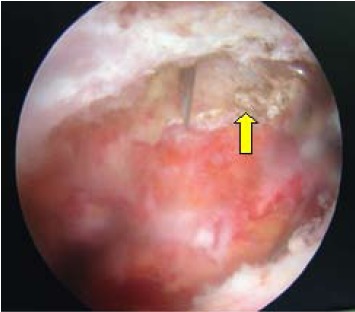# Arthroscopic Resection of The Distal Clavicle With Concomitant Subacromial Decompression: A Case Series

**DOI:** 10.5704/MOJ.1407.007

**Published:** 2014-07

**Authors:** HZ Chan, CL Ooi, MY Lim, EKS Ong, O Zulkiflee

**Affiliations:** Department of Orthopaedics, Hospital Pulau Pinang, Georgetown, Malaysia; Department of Orthopaedics, Hospital Pulau Pinang, Georgetown, Malaysia; Department of Orthopaedics, Hospital Pulau Pinang, Georgetown, Malaysia; Department of Orthopaedics, Hospital Pulau Pinang, Georgetown, Malaysia; Department of Orthopaedics, Hospital Pulau Pinang, Georgetown, Malaysia

## Abstract

Shoulder impingement syndrome and acromioclavicular joint osteoarthritis often occur simultaneously and easily missed. Kay et al. reported excellent results with combined arthroscopic subacromial decompression and resection of the distal end of the clavicle in patients with both disorders^1^. Arthroscopic treatment of these disorders produces more favourable results than open procedures. We report two patients who were not responding to conservative management and were treated with direct arthroscopic distal clavicle excision and subacromial decompression in single setting. Both patients gained good postoperative outcome in terms of pain score, function and strength improvement assessed objectively with visual analogue score (VAS) and University of California Los Angeles Score (UCLA).

**Key Words:**

Acromioclavicular joint arthritis, distal clavicle excision, Arthroscopy, Mumford operation.

## Introduction

Symptomatic acromioclavicular osteoarthritis and distal clavicle osteolysis can be treated effectively with both nonoperative and operative means. Non-operative treatments including physiotherapy, non-steroidal anti-inflammatory drugs (NSAIDS) and corticosteroid injection may help to relieve the symptoms. However, surgical ^l^ intervention is warranted when patients are unresponsive to conservative management. Resection of the distal clavicle, as described by Mumford ^2^, is a reliable surgical option in acromioclavicular joint (ACJ) osteoarthritis and shoulder impingement syndrome. Symptom improvement has been satisfactory in most reported series. Historically, distal clavicle resection has been performed using an open incision over the ACJ with detachment of the deltoid and trapezius muscles. Significant morbidity may follow with these open procedures. Wound infection, residual acromioclavicular joint instability, cosmetically unacceptable scar, postoperative shoulder weakness and stiffness are among the common complications reported from these open procedures ^3^.

## CASE REPORT

**Patient One**An active 64 year old lady with no previous history of trauma was referred for evaluation of persistent anterosuperior left shoulder pain for one year. The pain worsened when she tried to reach across the body or behind the back. She also experienced discomfort with rotational and overhead movements of the left shoulder. Physical examination revealed tenderness over the left acromioclavicular joint. Cross body adduction and active compression tests reproduced the symptoms at the acromioclavicular joint. Neer and Jobe impingement tests were positive. Plain radiographs revealed osteoarthritis of acromioclavicular joint with narrowing of joint space (Figure 1A). MRI of the shoulder joint on T2 weighted sequence revealed left acromioclavicular joint degenerative changes with proximal supraspinatus tendon impingement causing intrasubstance delamination tear (Figure 2). She was started with non-surgical treatment such as analgesia, rotator cuff and periscapular strengthening exercises. Initially, patient claimed that the pain resolved for a short period. Then, her symptoms recurred and worsened. Eventually, she was agreeable for concomitant arthroscopic distal clavicle resection and subacromial decompression surgery.

The patient was put on beach chair position. Through a standard posterior portal, the arthroscope was inserted to screen for underlying pathology. Meanwhile, another anterior portal was made via outside in technique with spinal needle, in line with the AC joint (to facilitate the AC joint resection later). Osteophytes were shaved through the anterior portal first, and then switched to posterior portal for bursectomy as this facilitated subacromial space viewing and subacromial decompression. Lateral portal was created with spinal needle, 3cm to the lateral of acromion edge. By viewing the scope from the lateral portal, resection of inferior part of AC joint was performed directly from the anterior portal. Further resection of AC joint, especially at the superior edge was achieved with the 70-degree scope to shave from anterior portal. Using the 70-degree scope from the standard lateral portal and also posterior portal, the entire AC joint could be viewed in detail which helped preserving the superior capsule and ligament. Finally, we switched the scope to the anterior portal for final checking for remnants of osteophytes and distal clavicle.

**Fig. 3: Patient demonstrating full range of movement of left shoulder without sign of impingement.**
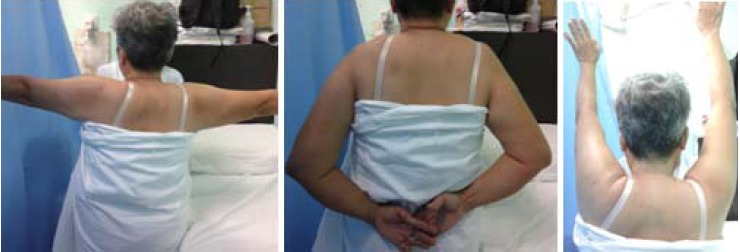


Postoperative, patient went through standard rehabilitation protocol, with early range of movement exercises from day one postoperatively. The shoulder was protected with arm sling for 7 to10 days. The patient returned to her normal activities at six weeks following surgery. Both VAS and UCLA scores showed improvements compared with preoperative findings. During follow up at six months, she remained pain free and achieved full range of movements of the shoulder without impingement signs (Figure 3). Plain radiographs demonstrated adequate distal clavicle resection (Figure 1B).

**Fig. 4: Preoperative X-ray showing not much acromioclavicular joint narrowing(A) Postoperative, distal clavicle resection.(B):**
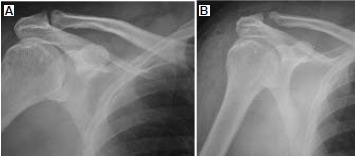


**Patient Two**A 54 year old male presented with a 4 year history of gradually worsening right anterosuperior shoulder pain. He had a history of a motor vehicle accident in 2009, sustaining direct trauma to his right shoulder. Arthroscopic debridement had been carried out in 2010 but the pain was only partially relieved. The patient had sought to relieve his pain with anti-inflammatory drugs, corticosteroid injection and physiotherapy but to little avail. Patient had limited range of movement of the right shoulder especially adduction. He experienced difficulty reaching for his wallet and tucking his shirt behind his back. Physical examination revealed healed previous arthroscopic portals. Positive tests included cross-body adduction and active compression, with pain located specifically at the acromioclavicular joint. There was no evidence of impingement signs or glenohumeral instability. Standard radiographs demonstrated maintenance of the acromioclavicular joint space, but marked hypertrophic degenerative changes (Figure 4A). MRI demonstrated right acromioclavicular joint arthritis and the rotator cuff appeared normal. The remaining bone, cartilage, and rotator cuff were normal in appearance (Figure 5). He also elected to proceed with arthroscopic distal clavicle resection. Similar operative technique as described before was used. Intraoperative finding was acromioclavicular joint arthritis and subacromial narrowing with supraspinatus impingement (Figure 6). However, the supraspinatus tendon was intact. In view of the findings, concomitant subacromial decompression was performed.

Postoperative radiographs demonstrated adequate bone resection and no obvious translation of the clavicle relative to the acromion (Figure 4B). Postoperative protection and physiotherapy was commenced. Patient returned to his usual activities at six weeks postoperatively. Full range of movement of shoulder was achieved at six months followup. There was no complaint of mechanical instability of the distal clavicle. Patient was satisfied with the result of the surgery, as he remained pain free. His VAS and UCLA scores had greatly improved.

## Discussion

Arthroscopic resection of the distal clavicle and subacromial decompression can avoid complications arising from the open method. Preserving the posterior and superior aspects of the acromioclavicular capsule avoids creating iatrogenic distal clavicular anteroposterior instability. Moreover, arthroscopic excision provides the advantage of evaluating glenohumeral joint at the time of surgery. Other shoulder joint pathology such as rotator cuff disease, loose bodies, labral tears and chondral injuries will not be missed. Hence, there will be only a single procedure needed to solve the patient’s shoulder problems. Arthroscopic subacromial decompression and arthroscopic resection of the acromioclavicular joint as separate procedures have been well documented. However, there is little documentation on the success rate of resection with concomitant subacromial decompression. In our case, we found excellent results with arthroscopic resection of the acromioclavicular joint and subacromial decompression in a single setting. Maintaining the integrity of the capsular attachments minimizes the amount of bleeding and trauma into the subacromial space, thereby decreasing postoperative pain while avoiding creating iatrogenic distal clavicular horizontal instability. When this procedure is performed on properly selected patients, there are fewer postoperative complications. Patients return to activities earlier while achieving similar long-term outcomes as the open procedure ^4^.

**Fig. 1: Preoperative plain radiograph of left shoulder showing degenerative changes of the cromioclavicular joint with narrowing of joint space(A). Postoperative radiograph of the same patient with distal clavicle resection(B).**
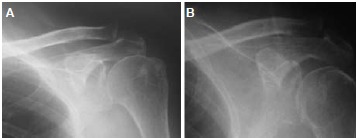


**Fig. 2: T2-weighted MRI left shoulder (coronal view) showing edema pattern beneath the left acromioclavicular joint (arrow) with arthritic changes.**
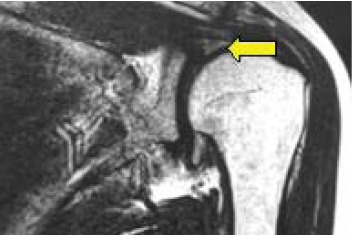


**Fig. 5: MRI right shoulder sagittal view showing marked arthropathic changes and the axial view with oedema below the acromioclavicular joint with supraspinatus impingement.**
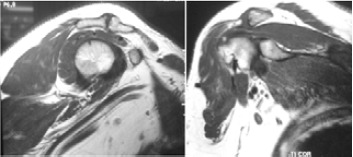


**Fig. 6: View from lateral portal of arthroscope: needle pierced through the acromioclavicular joint. Distal clavicle resected by using burr.**